# Statistical Analysis of the Indus Script Using *n*-Grams

**DOI:** 10.1371/journal.pone.0009506

**Published:** 2010-03-19

**Authors:** Nisha Yadav, Hrishikesh Joglekar, Rajesh P. N. Rao, Mayank N. Vahia, Ronojoy Adhikari, Iravatham Mahadevan

**Affiliations:** 1 Tata Institute of Fundamental Research, Colaba, Mumbai, India; 2 Mumbai, India; 3 Department of Computer Science and Engineering, University of Washington, Seattle, Washington, United States of America; 4 The Institute of Mathematical Sciences, Chennai, India; 5 Indus Research Centre, Roja Muthiah Research Library, Chennai, India; University of East Piedmont, Italy

## Abstract

The Indus script is one of the major undeciphered scripts of the ancient world. The small size of the corpus, the absence of bilingual texts, and the lack of definite knowledge of the underlying language has frustrated efforts at decipherment since the discovery of the remains of the Indus civilization. Building on previous statistical approaches, we apply the tools of statistical language processing, specifically *n*-gram Markov chains, to analyze the syntax of the Indus script. We find that unigrams follow a Zipf-Mandelbrot distribution. Text beginner and ender distributions are unequal, providing internal evidence for syntax. We see clear evidence of strong bigram correlations and extract significant pairs and triplets using a log-likelihood measure of association. Highly frequent pairs and triplets are not always highly significant. The model performance is evaluated using information-theoretic measures and cross-validation. The model can restore doubtfully read texts with an accuracy of about 75%. We find that a quadrigram Markov chain saturates information theoretic measures against a held-out corpus. Our work forms the basis for the development of a stochastic grammar which may be used to explore the syntax of the Indus script in greater detail.

## Introduction

The earliest urban civilization of the Indian subcontinent flourished in the valley of the river Indus and its surroundings during the Bronze Age. At its peak, in the period between 2600 BCE and 1900 BCE [1], it covered approximately a million square kilometers [2], making it the largest urban civilization of the ancient world. The remains of the civilization were first found in Harappa and, following historical convention, is called the Harappan civilization.

**Figure 1:**
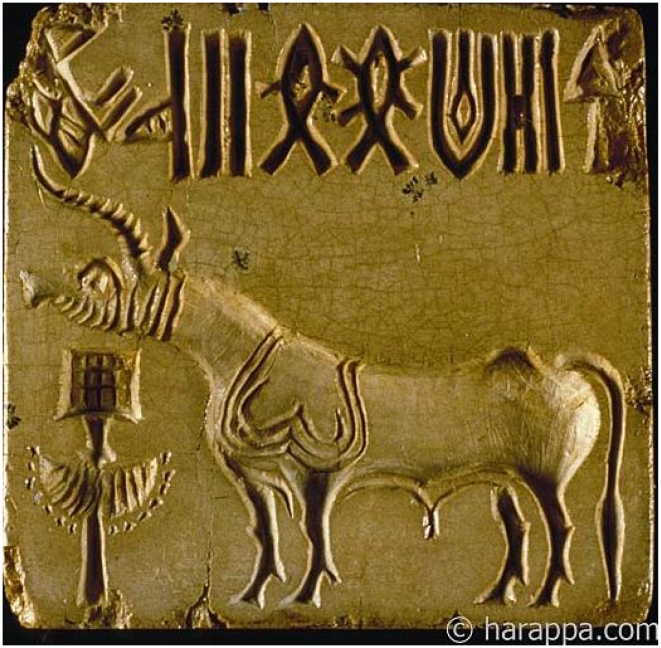
An example of an Indus seal. It shows the three typical components: the Indus script at the top, a field symbol (an animal) in the middle, and a decorated object at the bottom left (Copyright Harappa Archaeological Research Project/J.M. Kenoyer, Courtesy Dept. of Archeology and Museums, Govt. of Pakistan). Here, since the script is embossed on a seal, it is to be read from the left to the right, whereas on the sealing, which are impressions of the seal, it is read from the right to the left. For the most part, the seals are typically between 1 to 2 square inches in size.

The Indus people used a script, which has mainly survived on seals (see Fig. 1 for an example), pottery, and other artifacts made of durable materials such as stone, terracotta and copper. The script is yet to be deciphered. The script occurs usually in short texts, numbering not more than 14 signs in a single line of text. Around 400 distinct signs have been identified [3], [4], though Wells identifies up to 676 distinct signs [5]. The total number of texts is about 3000. Obstacles to the decipherment of the sign system include the paucity of long texts, the absence of bilingual text, and the lack of any definite knowledge of the underlying language(s) the script may have expressed.

The Indus script remains controversial, with contested claims of decipherment. The main methodological difficulty in attempting any interpretation of the script is that, due to the paucity of information on the context of the writing, one is perforce required to make an assumption regarding the content of the script. This leads to a profusion of interpretations, which are often not even falsifiable. The range of opinion on what the script encodes varies from an Indo-Aryan language [6] and a Dravidian language [4] to a purely numerical system [7]. There is no consensus on any of the above interpretations.

A more objective approach, not requiring *a priori* assumptions, is the method of statistical analysis. The method involves identification of patterns through counting. While such an approach cannot shed light on the semantics of the script, it can reveal important features of its syntax. Research on the Indus script using the statistical approach was initiated by Knorozov and his team in 1965, further developed by Parpola and collaborators in 1969 (for review of various attempts see [8]–[10]), continued by Siromoney [11] in the 1980s and followed up more recently by us [12]–[15].

In this article, we apply the technique of *n*-gram modeling [16], [17] for a thorough statistical analysis of sequences in the Indus script. This technique finds widespread use in the analysis of sequences, be they letters or words in a natural language, the base pairs in the genetic code, or the notes in a musical score. This generality is possible because *n*-gram models are indifferent to the semantic content of the units or tokens (the words, the letters, the base pairs or the notes) making up the sequence but, nonetheless, reveal the syntax, if any, that the sequences follow. The *n*-gram approach, then, provides a framework in which the Indus script can be studied without making any *a priori* assumptions. Our previous work explored some applications of bigrams (an *n*-gram model with n = 2) to analyze the Indus script [14], [15]. This paper presents further results for the bigram model and extends the analysis to higher order *n*-grams.

## Results and Discussion

### Empirical Analysis

**Figure 2:**
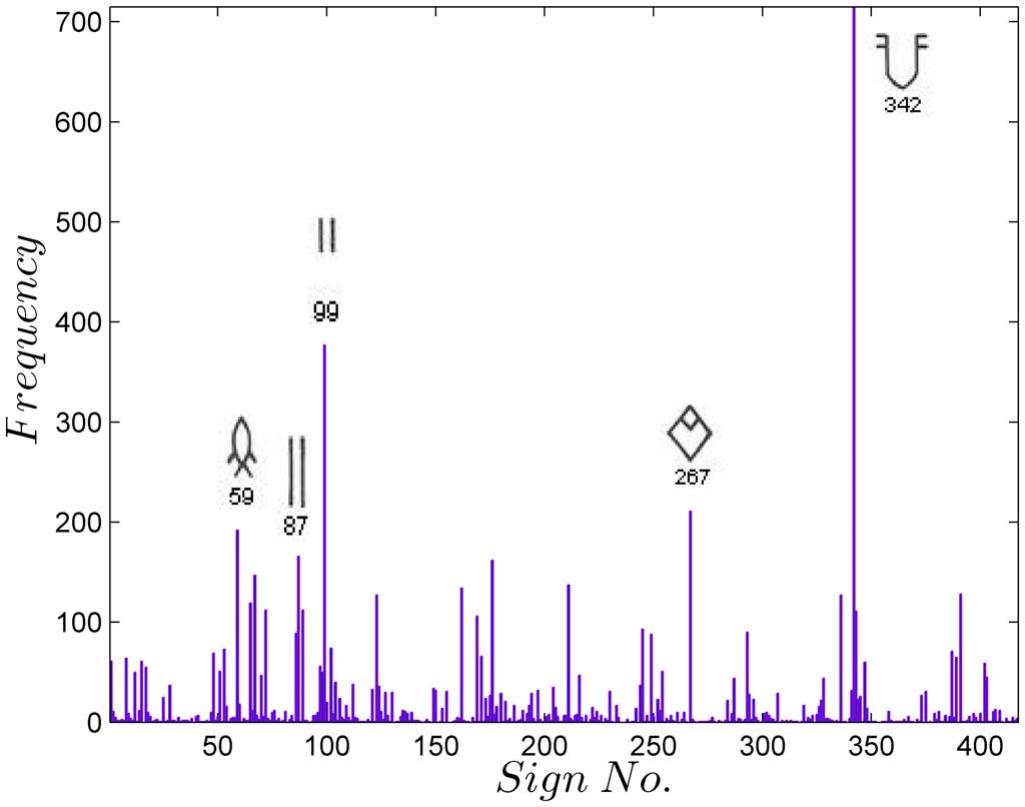
Frequency distribution of individual signs in the EBUDS corpus. The five most common signs are shown alongside the frequency bars. The relative frequency distribution does not change significantly between EBUDS and M77 corpora.

Statistical analysis of the Indus script requires a standard corpus. Three major corpora of the Indus texts, by Mahadevan [3], Parpola [4] and Wells [5], are available. We use the electronic concordance of Mahadevan, henceforth referred to as M77, which records 417 unique signs in 3573 lines of 2906 texts (see Materials and Methods for details). We first present the results of an empirical statistical analysis of the EBUDS corpus. EBUDS is a filtered corpus created from M77 to remove duplicates and ambiguities (see Materials and Methods for details). Fig. 2 shows the frequency distribution of signs in EBUDS. The sign corresponding to 342 in M77, is the most frequent sign, followed by signs 99, 267, and 59. The relative frequencies have no significant change in the M77 and EBUDS corpora.

**Figure 3:**
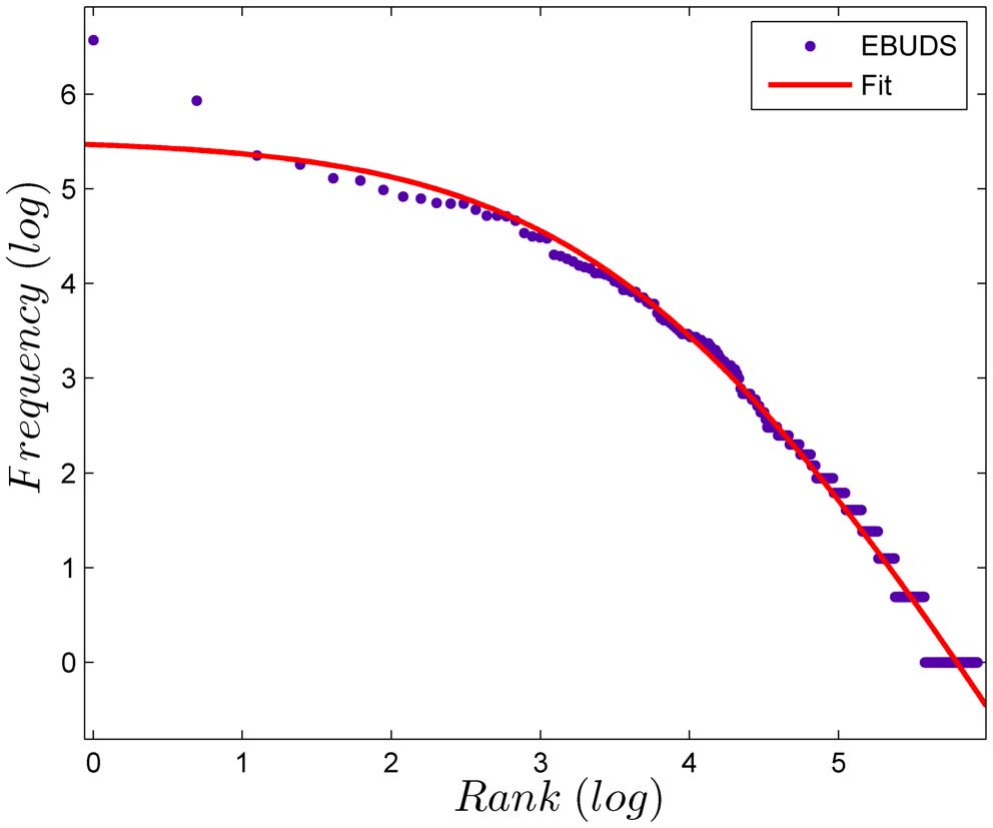
Rank-ordered frequency distribution of signs ƒ*_r_* plotted against the rank *r* for the EBUDS corpus. The data can be fit well by the Zipf-Mandelbrot law, 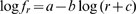. For *c* = *0* and *b* = 1, this reduces to Zipf's Law, 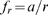. Both these laws are used to fit the frequency distribution of words in linguistic corpora. Our fitted values are *a* = 15.39, *b = 2.59* and *c* = 44.47. For English (the Brown Corpus), *a* = 12.43, *b* = 1.15 and *c* = 100 [16].

The same data can be plotted as a rank-ordered frequency distribution. The most frequent sign is given rank *r* = 1 and its frequency is denoted by ƒ_1_, the next most frequent sign is given rank *r* = 2 and its frequency is denoted as ƒ_2_ and so on, till all signs are exhausted. The rank-ordered frequency ƒ*_r_* is then plotted against the rank *r*, on double logarithmic axes, as shown in Fig. 3. The data can be fit very well to the Zipf-Mandelbrot law, 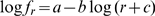 [16]. This statistical regularity in word distributions is found across a wide range of languages [16], [18]. Mandelbrot [19] has shown that the Zipf-Mandelbrot law appears as a consequence of linguistic evolution that tends to maximize information per word under the constraint of constant effort, or equivalently, to minimize effort per word under the constraint of constant information. Thus the Zipf-Mandelbrot law emerges, in this derivation, as a plausible necessary feature of linguistic tokens. Clearly, since distributions of city sizes, incomes and several other quantities also follow a Zipf distribution, the presence of a Zipf-Mandelbrot distribution is not sufficient to declare a sequence as linguistic. It is significant that the distribution of individual tokens in the Indus script follows a Zipf-Mandelbrot distribution. Qualitatively, a distribution which follows the Zipf-Mandelbrot law has a small number of tokens which contribute to the bulk of the distribution, but also a large number of rare tokens which contribute to a long tail. To emphasize this point, it is estimated that English has approximately a million words, though a college graduate might know only between 60,000 to 75,000 of these, and yet be a competent user of the language [20]. The Indus script seems to follow the same pattern, with a small number of signs accounting for the majority of usage, but with a large number of signs which are used infrequently.

**Figure 4:**
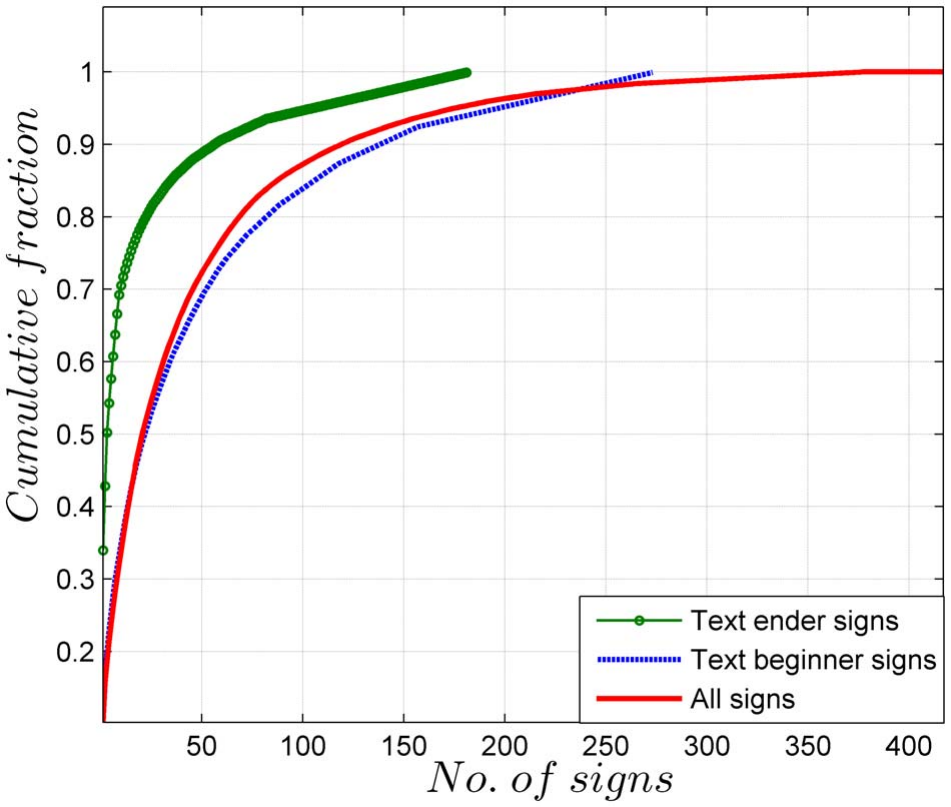
Cumulative frequency distribution of all signs, only text beginners, and only text enders in the EBUDS corpus. Approximately 69 signs account for 80% of the corpus. The script has a large number of signs which are used infrequently. The cumulative distributions for text beginners and text enders show an asymmetry, with only 23 signs accounting for 80% of all text enders, while 82 signs account for 80% of all text beginners. This is clear evidence of an underlying logic in the sign usage.

To further follow up this point, we plot the cumulative frequency distribution of the signs in EBUDS in Fig. 4. As can be seen from the graph, 69 signs account for about 80% of EBUDS and the most frequent sign (342) alone accounts for 10% of EBUDS. This observation is consistent with previous analysis by Mahadevan for M77 corpus [3].

In the same graph, we plot the cumulative distribution of text beginners and text enders. Here, an interesting asymmetry is evident: 82 text beginners account for about 80% of the text beginner usage, but only 23 text enders are needed to account for the same percentage of text ender usage. Since the possible set of text beginners and text enders can include any of the 417 signs, the numbers above indicate that both text beginners and text enders are well-defined, with text enders being more strictly defined than text beginners. This indicates the presence of syntax in the writing.

The analysis above has only been concerned with frequency distribution of single signs. We may extend the analysis to sign pairs, sign triplets and so on, as in our earlier work [12], [13], [15]. This allows one to explore the order and correlations between the signs, which are the manifestations of syntax. Below, we explore a general *n*-gram Markov model to study how sign order and sign correlations can provide insights into the syntax of the Indus script. Throughout, we use “correlated” to imply that the joint distribution of the variables cannot be factored into products of individual distributions. This applies not only to bigrams with *n* = 2 but also to the general *n*-gram with n>2. We assume the Markov chain to be stationary.

### *n*-Gram Model for the Indus Script

An *n*-gram model can identify the correlations that exist between tokens 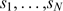 in a sequence *S_N_* of *N* tokens. Conditional probabilities form the core of an *n*-gram model. Specifically, for a string 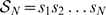 the n-gram model is a specification of conditional probabilities of the form 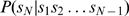, quantifying the probability that the previous *N*−1 signs of the sub string 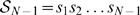 is followed by the sign 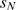. Given the *n*-gram conditional probability, and the relation between joint and conditional probabilities 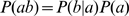, the probability of the string *S_N_* can be written as,
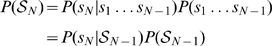
(1)Recursively applying 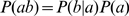 to the rightmost terms, we obtain the probability as a product over conditional probabilities
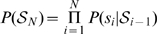
(2)In the above, it is understood that S_0_ = # is a special token indicating the start of the string. Note that the above expression is an identity that follows from the basic rules of probability and contains no approximations. As an example, the probability of a string of length three, 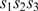, is given as a product of trigram, bigram and unigram probabilities
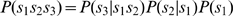
(3)Clearly, for an *n*-gram model to be tractable, only a finite number of such probabilities can be retained. In the simplest bigram model, all correlations beyond the preceding sign are discarded, so that
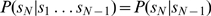
(4)In a trigram model, all correlations beyond two preceding signs are discarded, so that
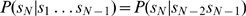
(5)In a general *n*-gram model, all correlations beyond the (*n−1*) preceding signs are discarded. An *n* -gram model can then be thought of as an (*n*−1)*^th^* order Markov chain in a state space consisting of the signs *s_i_*. The introduction of the Markov assumption is the main approximation in *n*-gram models. *n*-grams were first used by Markov [21] to analyze the probability of a consonant following a vowel in Pushkin's *Eugene Onegin*. Shannon applied word *n*-grams to model sentences in English text and with *n* = 4 obtained sentences which have a remarkable syntactic similarity to English [22]. Since then, *n*-gram models have found wide use in many fields where sequences are to be analyzed, including bioinformatics, speech processing and music. The theory and applications of *n*-grams are described in several textbooks [16], [17]. Our method of obtaining probabilities from counts and the use of smoothing and backoff to account for unseen *n*-grams is described in Materials and Methods. The measures we use for evaluating the *n*-gram model and the tests we use to assign a statistical significance to the correlations are discussed below.

In any *n*-gram study, a maximum value of *n* has to be chosen in the interest of tractability, beyond which correlations are discarded. This can be done in an empirical fashion, balancing the needs of accuracy and computational complexity, using measures from information theory which discriminate between *n*-grams models with increasing *n* [16], [17], or by more sophisticated methods like the Akaike Information Criterion which directly provides an optimal value for *n* [23].

In previous work [12], it was shown that bigram and trigram frequencies in the EBUDS corpus differ significantly from frequencies expected from a Bernoulli scheme. The small size of the corpus limits the ability to assess significance of quadrigrams and beyond, when using the method in [12]. In our subsequent work [13] it has been shown that 88% of the texts of length 5 and above can be segmented using frequent unigrams, bigrams, trigrams and quadrigrams and complete texts of length 2, 3 and 4. Moreover, frequent bigrams or texts of length 2 alone account for 52% of the segmented corpus. Thus the bulk of the corpus can be segmented with *n*-grams with *n* not exceeding 4, and almost half the corpus can be segmented into bigrams alone.

Here, we use cross-entropy and perplexity, discussed in detail below, to measure how well *n*-gram models with varying *n* capture the syntax in the corpus. We have modeled the EBUDS corpus with successive orders of Markov chains starting with *n* = 1 to *n* = 5 for calculating perplexity. Our evaluation methodology involves partitioning the corpus into a training set (from which the n-gram probabilities are learnt) and a test set (on which the *n*-gram probabilities are evaluated). The test set is commonly called a held-out corpus. We find that the perplexity monotonically decreases as *n* ranges from 1 to 3 (corresponding to unigram, bigram and trigram correlations), but then saturates beyond *n* = 4 (corresponding to quadrigram and higher correlations, see Table 1). This is consistent with our earlier work [12], [13] where syntactic units consisting at most of quadrigrams were identified on the basis of frequency and segmentation analysis.

**Table 1:** Perplexity and the *n*-gram cross entropy *H_n_(Q,P)* for the EBUDS corpus.

*n*	1	2	3	4	5
Perplexity ( 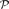 )	68.82	26.69	26.09	25.26	25.26
*H_n_(Q,P)*	6.10	4.74	4.71	4.66	4.66

The perplexity reduces dramatically when bigram correlations are included, has a small but significant reduction with trigram correlations, but then saturates beyond quadrigram correlations. This indicates that a bigram model captures a significant portion of the syntax.

From the differential reduction in perplexity with increase in model order, it is clear that the most significant correlations are due to bigrams, with somewhat modest trigram correlations, and almost negligible quadrigram correlations. This appears reasonable, given that the mean length of the Indus texts is about five tokens. Here we present detailed analysis of bigram probabilities, and representative results for trigrams. The main conclusions that we draw in this paper on the structure of the script are expected to remain broadly unaltered with the inclusion of trigram and quadrigram correlations. The role of higher order correlations will be more fully explored in forthcoming work.

### Analysis of Bigrams

We now present the results of a bigram analysis of the sequence of signs in the EBUDS corpus. In a bigram model, it is assumed that the probability 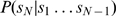 depends only on the immediately preceding sign and is the same as 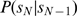. The bigram model is fully specified by the unigram probabilities 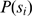 and the bigram conditional probabilities 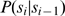.

We introduce two additional tokens # and $ which indicate the beginning and end of a text respectively. By convention, the unigram probability for the start token is unity, 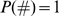, since every text must begin with #. The probability of sign *a* being a beginner is then 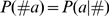, since 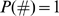. The probability of sign *a* being an ender is 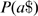.

**Figure 5:**
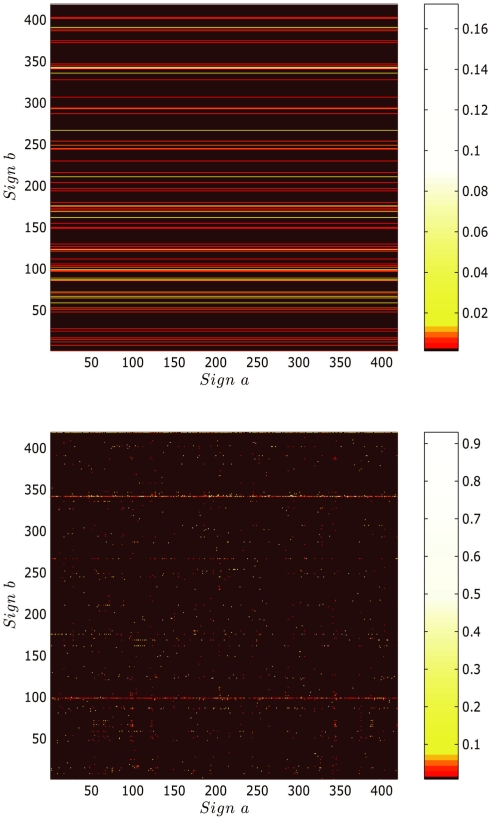
Bigram probability *P*(*b*|*a*) for a random distribution with no correlations amongst the signs (above) and for the EBUDS corpus (below). Horizontal lines in the upper matrix imply that the conditional probability of a sign *b* following a sign *a* is equal to probability of sign *b* itself. The bigram probability *P*(*b|a*) after Witten-Bell smoothing is shown in the lower plot. The difference between the two matrices indicates the presence of correlations in the texts.

The two plots in Fig. 5 compare the bigram conditional probabilities in the absence of correlations (such that 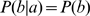 is just the unigram probability) with the bigram conditional probabilities for EBUDS corpus after Witten-Bell smoothing (see Materials and Methods). If there is no correlation between *b* and *a*, we expect 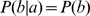, that is, the conditional probability of *b* is identical to the marginal probability. We show this marginal probability in the first plot of Fig. 5. In the second plot of Fig. 5 we show the matrix of bigram conditional probabilities 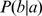 that sign *b* follows sign *a* in the corpus. There are significant differences between the two plots. This indicates the presence of correlations in the script and the necessity of going beyond unigrams to model the script.

**Figure 6:**
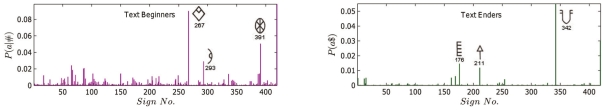
Probability *P*(*a*|#) of a sign 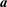 following the start token # (text beginners) and probability *P*(*a*$) of sign *a* preceding the end token $ (text enders). This is extracted from bigram matrix 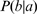 with Witten-Bell smoothing. Text beginners with a significant probability are more numerous than text enders at the same threshold of probability.

In Fig. 6 we show the text beginner and text ender sign probability distributions. This confirms our earlier conclusion (Fig. 4), based on raw counts, that text enders are more strictly defined than text beginners.

**Figure 7:**
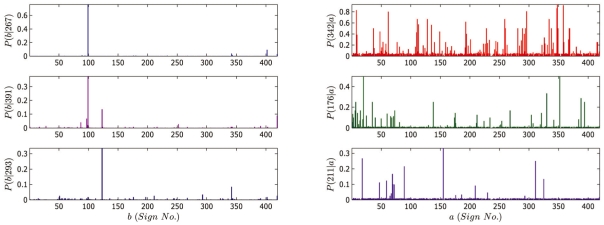
Conditional probability plots for text beginners 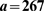, 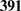, 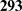 followed by sign 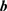 and for texts enders 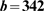, 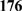, 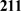 preceded by sign 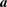 from bigram matrix *P*(*b*|*a*) with Witten-Bell smoothing. Text beginners are more selective in terms of the number of signs which can follow them than text enders, which can have a large number of signs preceding them.

We can further analyze the nature of correlations of a sign with other signs preceding or following it using the results of bigram analysis. As an example, we explore the correlations of the three most frequent text beginners (sign numbers 267, 391, and 293) and the three most frequent text enders (sign numbers 342, 176 and 211) shown in Fig. 6 with other signs. It can be inferred from the plots of conditional probabilities in Fig. 7, that is, 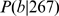, 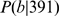 and 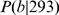 for the text beginners and 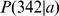, 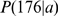 and 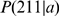 for the text enders, that the text beginners 267, 391 and 293 are more selective in terms of the number of signs which can follow them in comparison to the text enders 342, 176 and 211 which can be preceded by relatively larger number of signs. Thus, there is greater flexibility for signs preceding the text enders than the signs which tend to follow the text beginners.

**Figure 8:**
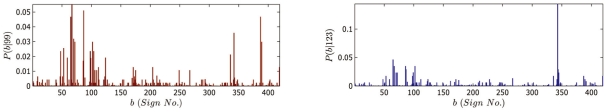
Conditional probability plots for sign *b* following text beginners *a* = 99 and *a* = 123. The number of signs following the signs 99 and 123 is greater than the number of signs following text beginners 267, 391 and 293 (Fig. 7).

We can gain further insights into the sequential structure of the texts by examining the signs 99 and 123 which most often follow the text beginners. The number of signs which follow 99 and 123 is quite similar to the number of signs that precede the text enders 342, 176 and 211 though in reverse direction (see plots of 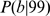 and 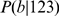 in Fig. 8). This helps us in finding the weaker and stronger junctions in the texts as shown in [13] where this information was used in segmenting the long texts into smaller segments.

The diagonal elements 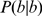 of the matrix of bigram probabilities are the probabilities of sign pairs with same signs. The most frequent sign pairs with repeating signs in the corpus are (153,153) and (245,245).

To quantitatively assess the significance of correlations, and to obtain statistically significant sign pairs, we need to test the null hypothesis that signs 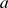 and 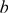 are independent. In Table 2 we give the most frequent sign pairs as well as the ones which are statistically most significant. Here, we enumerate the 20 most significant sign pairs on the basis of log-likelihood ratio measure of association for bigrams [24]. It is interesting to note that the most significant sign pairs are not always the most frequent ones (given in the first column of Table 2). Such a conclusion has also been arrived at using an independent method of evaluating significant pairs [25]. An exhaustive analysis of sign correlations, using several measures of association and including significant bigrams, trigrams and quadrigrams, will be forthcoming.

**Table 2:** Significant sign pairs from the log-likelihood ratio (LLR) measure of association for bigrams.

Sign Pair	Rank	Frequency	Significant	Rank	LLR Value
	(Naive)	(EBUDS)	Sign Pair	(LLR)	
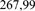	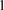	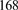	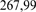	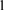	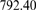
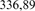	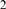	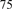	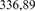	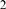	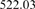
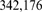	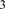	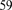	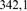	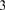	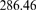
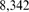	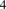	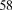	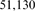	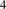	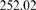
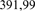	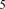	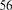	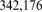	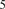	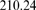
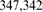	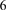	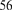	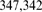	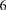	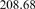
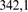	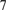	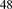	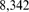	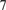	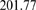
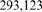	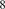	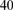	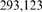	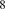	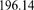
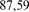	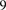	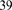	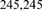	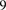	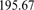
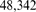	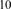	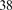	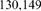	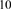	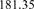
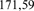	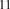	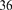	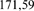	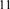	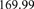
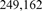	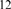	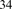	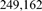	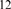	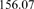
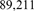	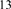	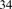	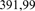	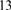	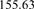
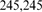	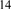	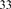	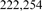	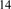	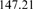
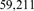	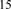	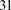	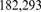	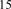	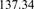
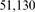	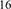	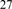	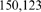	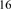	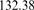
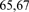	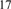	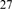	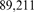	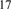	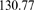
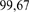	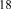	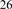	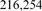	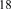	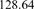
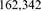	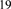	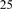	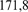	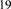	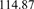
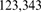	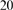	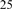	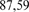	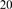	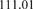

The 20 most frequent sign pairs (first column) are compared with the 20 most significant sign pairs (third column). The most frequent sign pairs are not necessarily the most significant sign pairs, as measured by the log-likelihood ratio measure of association.

**Figure 9:**
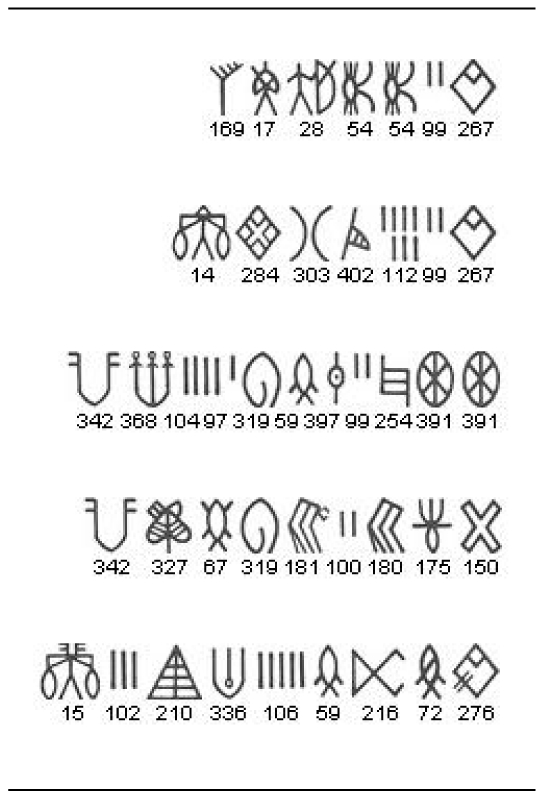
Examples of texts generated by the bigram model. The texts are to be read from the right to the left. Some of the texts generated by the model occur in the corpus.

The bigram model can be used to generate texts according to the Markov chain defined by the unigram and bigram probabilities. In Fig. 9 we show examples of texts generated by the bigram model. The evaluation of the performance of the model is discussed in Materials and Methods.

To summarize, the results above show that it is necessary to go beyond unigrams in modeling the EBUDS corpus, that there is significant structure in the bigram probabilities, and that the bigram probabilities themselves are statistically significant.

### Analysis of Trigrams

**Figure 10:**
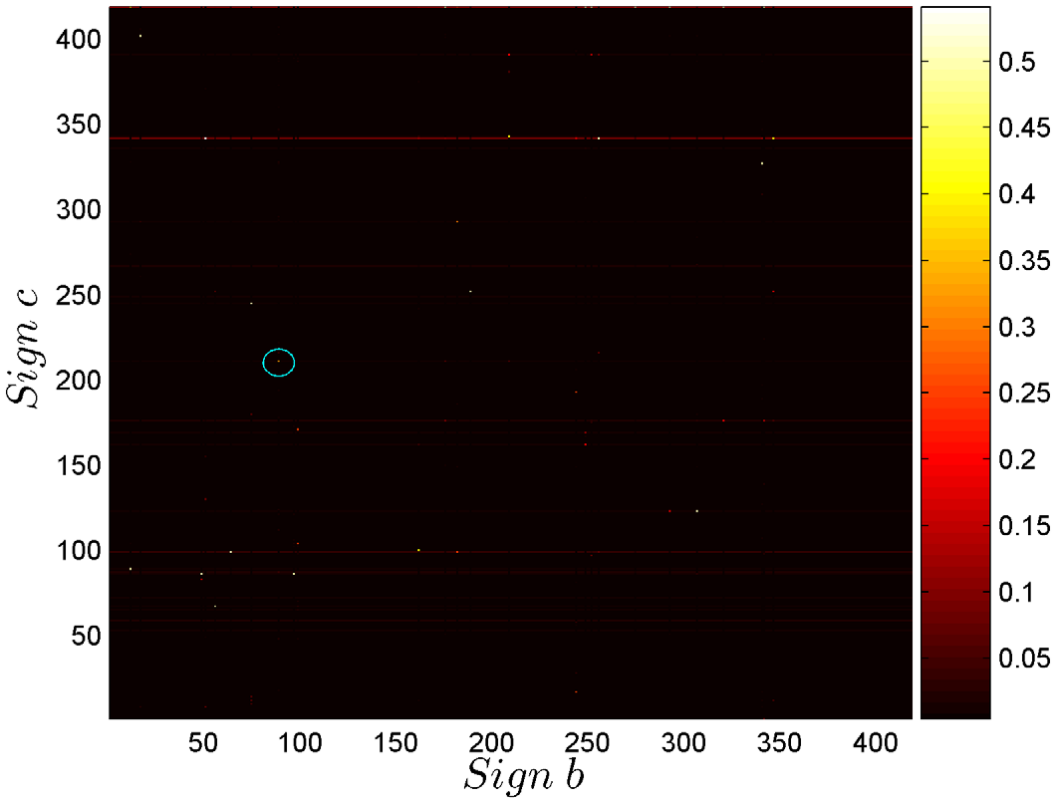
A section of the trigram matrix. Trigram conditional probability *P*(c|*ab*), with a = 336, the most frequent triplet being 336,89,211 (circled in the plot). This gives the trigram conditional probability of all strings of the form 336,*b*,*c*.

The general *n*-gram model allows us to systematically go beyond bigrams. In a trigram model, it is assumed that the probability 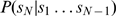 depends only on the two immediately preceding signs and is the same as 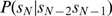. The trigram model is fully specified by the unigram probabilities 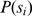, the bigram conditional probabilities 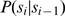, and the trigram conditional probabilities 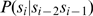. While the unigram and bigram conditional probabilities can be conveniently displayed as a graph and as a matrix, the trigram probability, which is a three-dimensional array, is not as easily displayed. It is possible to study sections of the three-dimensional array by fixing one of the three indices of the trigram conditional probability, and studying the variation of the remaining two. In Fig. 10 we have plotted a section of the trigram conditional probability 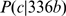, choosing *a* = 336, as the most frequent triplet is 336,89,211. This gives the trigram conditional probability of all strings of the form 336,*b*,*c*. Exhaustive studies of the type done for bigrams can also be performed, but we leave reporting this for future work.

Significant trigrams can also be assessed from the trigram conditional probabilities. Here, there are four possible null hypotheses that can be tested, namely that of complete independence 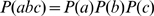, and that of pairwise independence 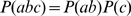 or 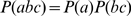 or 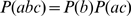. Since bigram correlations have already been established, assessing significant trigrams based on the first null hypothesis is redundant. Instead, we test for pairwise independence using the same log-likelihood ratio measure of association used for bigrams [24]. The 20 most significant triplets using the null hypothesis, 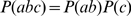, is displayed in Table 3. As in the case of bigrams, the most frequent sign triplets are not the most significant ones. Also, like the sign pairs the sign triplets also seem to have a preferred location within the texts [12].

**Table 3:** Significant sign triplets from the log-likelihood ratio (LLR) measure of association for trigrams.

Sign Triplet	Rank	Frequency	Significant	Rank	LLR Value
	(Naive)	(EBUDS)	Sign Triplet	(LLR)	
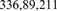	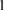	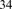	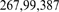	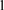	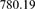
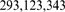	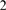	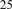	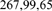	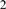	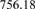
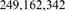	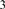	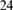	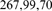	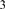	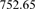
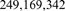	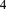	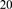	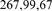	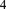	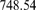
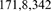	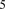	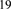	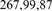	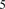	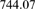
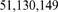	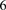	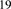	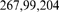	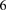	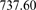
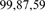	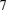	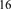	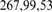	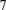	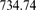
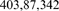	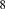	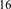	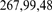	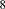	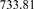
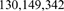	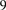	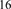	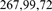	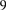	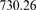
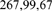	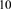	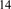	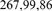	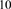	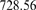
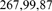	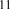	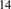	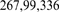	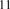	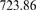
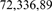	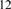	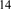	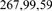	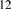	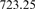
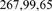	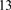	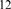	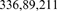	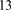	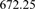
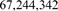	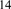	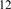	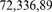	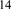	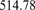
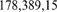	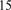	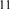	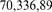	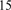	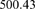
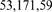	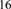	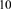	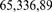	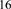	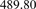
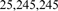	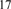	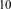	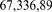	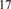	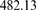
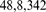	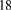	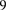	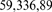	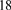	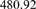
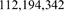	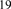	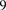	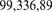	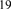	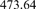
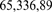	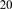	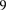	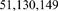	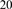	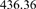

We use the null hypothesis, 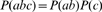. The 20 most frequent sign triplets (first column) are compared with the 20 most significant sign triplets (third column). The most frequent sign triplets are not necessarily the most significant sign triplets, as measured by the log-likelihood ratio measure of association.

### Significance of Correlations

Information theoretic measures (see Materials and Methods for details) are commonly used to measure the goodness of *n*-gram models and to assess the significance of correlations between tokens. Here, we supplement our previous analysis of the bigrams and trigrams with information theoretic measures such as the entropy, mutual information (see Materials and Methods for details) and perplexity.

In Table 4 we compare the unigram entropy and bigram mutual information of the corpus with that of a completely random sequence with no correlations. For this, the probability of signs is uniform *P(a)* = 1/377 (since only 377 signs out of 417 appear in EBUDS) and the joint probability is 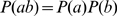. This gives an entropy of 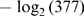 = 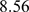 and a vanishing mutual information. In contrast, the unigram entropy of the EBUDS corpus is 6.68 and the bigram mutual information is 2.24. This also points to the presence of correlations, but the difference between the entropy and mutual information also indicates that there is flexibility in the sign usage, and the probability of a sign is not completely determined by the preceding sign.

**Table 4:** The entropy and mutual information of the EBUDS corpus.

Measure	Random	EBUDS
Entropy (H)	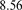	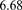
Mutual information (I)	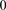	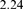

The entropy is smaller than a random equiprobable sequence of 417 signs. The mutual information is non-zero, indicating the presence of correlations between consecutive signs.

The main goal of *n*-gram analysis is to construct a good model for the probability of sequences in a corpus. The cross-entropy is a useful metric in evaluating the performance of *n*-gram models with different n. For a true distribution *Q(a)* and its estimate *P(a)*, the cross entropy is defined as,
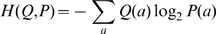
(6)The cross-entropy is minimum when the true and estimated probability distributions coincide, *Q(a)* = *P(a)*. As the model accuracy increases, the cross entropy H(Q,P) approaches the true entropy H(Q) of the corpus. The perplexity 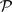, which is the measure commonly used in the natural language processing, is the exponential of the cross-entropy,
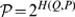
(7)The true probability distribution 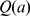 is not known, but it can be shown [16], [17] that for a corpus of size *M* obtained from a stationary and ergodic chain, the cross-entropy is given by the limit,
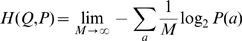
(8)


The previous formula does not require knowledge of *Q(a)*, and can then be used to give an estimate of the cross-entropy for a large, but finite, corpus. The relation has obvious generalizations to joint probability distributions 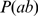, 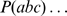, of bigrams, trigrams and higher *n*-grams. We denote the *n*-gram cross-entropy by 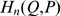.

We measure the cross-entropy against a held-out portion of the EBUDS corpus. The perplexity is reduced considerably when bigram correlations are taken into account. This is consistent with the previous analysis using entropy and mutual information. We have also evaluated the perplexity for trigram and quadrigram models, and this shows a monotonic reduction in perplexity as shown in Table 1. This implies that correlations beyond the preceding sign are important, though the most important correlations comes from the preceding sign. The perplexity of the bigram model is 26.69 which is significantly lower than that of unigram model which equals 68.82. As discussed in the beginning of this section, this motivates our choice of retaining only bigram correlations for the present study. From the differential reduction in perplexity, it is fair to conclude that the bulk of the correlations are captured in bigrams. Applications based on bigram correlations alone can therefore be expected to be reasonably accurate. The evaluation of the bigram model using cross-validation is discussed below.

### Restoring Illegible Signs

**Figure 11:**
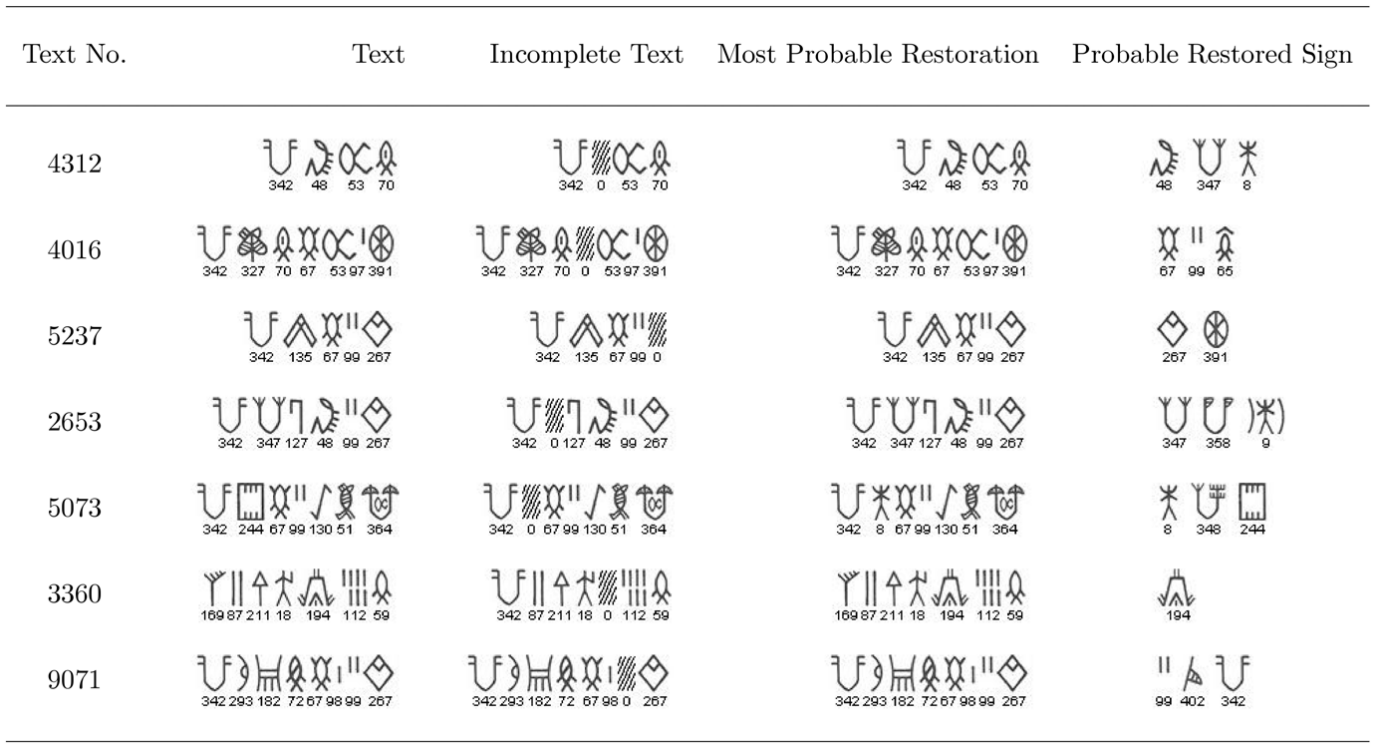
Suggested restoration of signs missing from texts. The last column lists the suggested restorations in decreasing order of probability (Left to Right).

**Figure 12:**
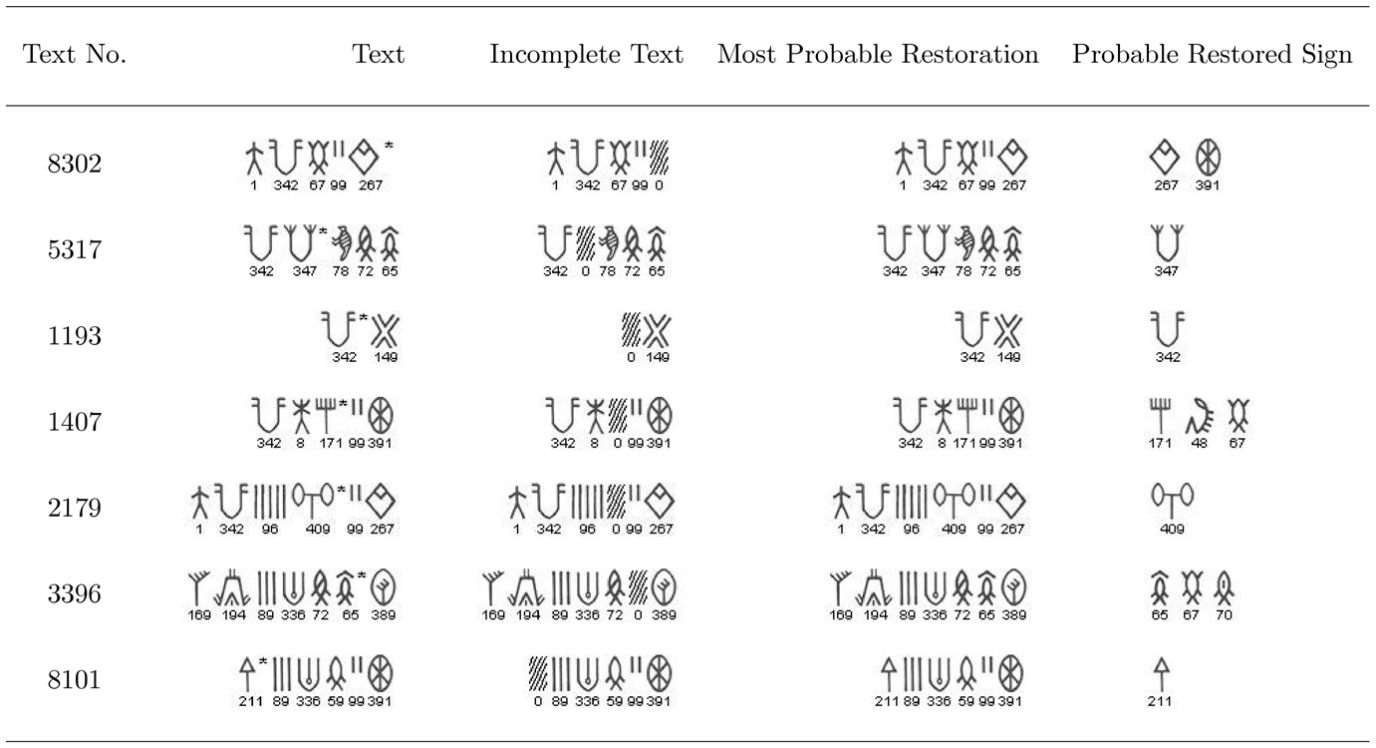
Suggested restoration of doubtfully read signs in the texts of M77 corpus. The last column lists the suggested restorations in decreasing order of probability (Left to Right). The signs with asterisk sign at the top right are the doubtfully read signs which are being restored using the bigram model.

An important practical use of the bigram model, first suggested in [15], is to restore signs which are not legible in the corpus due to damage or other reasons. We can use the bigram model to evaluate the probability of a suggested restoration, and choose the restoration with the highest probability. For example, consider the three sign text 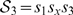 in which the middle sign 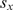 is illegible. We use the bigram model to evaluate the probability of the string for different choices of 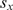 by
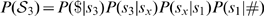
(9)The most probable sign with which to restore the text is, then, the maximum of this probability over all possible signs 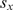. Since there are 417 possible signs, this can be accomplished by a direct enumeration. When the number of illegible signs is more, the space over which the maximization needs to be done grows rapidly. With *p* illegible signs, there are 417*p* values from which to pick a maximum. In such instances, instead of a direct search, a dynamic programming algorithm may be applied. Here, we use the Viterbi algorithm [16] for finding the most probable state sequence for a given observation in a hidden Markov model, suitably modified to make it applicable to a Markov chain, to find most probable sequence of signs. Our results for sign restorations are summarized in Fig. 11. We list the original text, a randomly chosen deletion for that text, the most probable restoration, and the next probable restorations obtained using the bigram model. We see that in all cases, the bigram model is successful in reproducing the deleted sign. This gives us confidence that the bigram model can be used to suggest restorations of illegible signs in various corpora. Fig. 12 gives few examples of how the model can be used for restoration of doubtfully read signs in the texts of M77 corpus.

**Figure 13:**
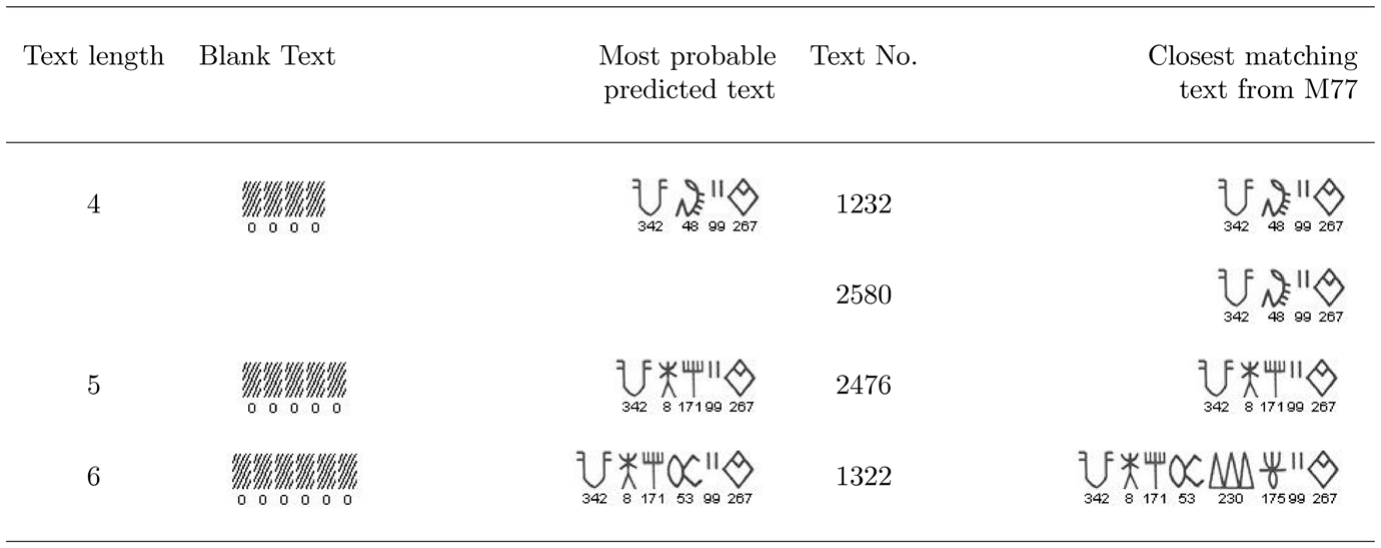
The most probable texts of length 4, 5 and 6 predicted by the model. Note that exact instances of the predicted texts are present in the corpus for the 4-sign and 5-sign texts. For the 6-sign text, the same sequence, but with 2insertions, is found in the corpus.

We can also use the model to find most probable texts of various lengths. In Fig. 13 we reproduce the most probable texts of length 4,5 and 6 as predicted by the bigram model. There are exact instances of the most probable texts of length 4,5 and slight variants of most probable text of length 6 in the M77 corpus.

### Model Performance Evaluation by Cross-Validation

The restoration algorithm also provides another measure of the model performance by cross-validation. The corpus is divided into a training set, from which the probabilities are estimated, and a test set, on which the model is evaluated. The measure of goodness of the model calculated is sensitivity defined as
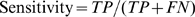
(10)where *TP* is the count of true positives and *FN* is the count of false negatives. The ratio of training to test set size used is 80∶20. We divide EBUDS into 5 equal parts i.e. *P*1, *P*2, *P* 3, *P4* and *P5* each being one-fifth of the corpus. We start with the first part, selecting that to be the test set and concatenate the remaining parts to form the first training set. We use the training set to learn the parameters of the model and use the test set to evaluate the goodness of the model. We drop out signs randomly from the test set and ask the model to fill in the dropped signs. We then count the number of true positives, that is, the number of times the predicted signs match with the signs under 90% area of the cumulative probability curve; otherwise, they are considered false negatives. This is repeated 100 times with the first test set and training set.

**Figure 14:**
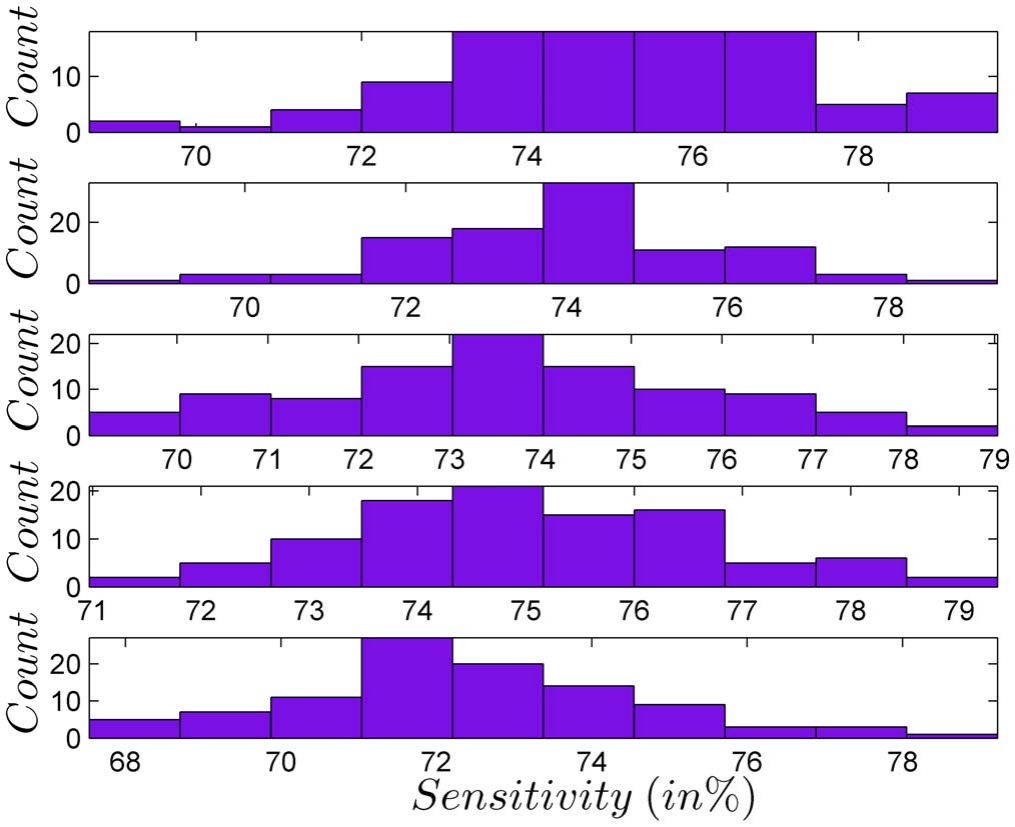
Sensitivity of the bigram model taking all signs under 90% area of the cumulative probability curve as true positives. The five plots are for five different sets of test and training sets of EBUDS as given in Table 5.

The cross-validation test described above is repeated by taking each of the five equal parts of EBUDS as the test set and concatenating the remaining part as the training set. The results are shown in Table 5 and Fig. 14. As can be seen from the plots, the sensitivity of the model, considering all signs under 90% area of the cumulative probability curve as true positives, is 74%.

**Table 5:** Mean sensitivity (in %) with standard deviation of the model predicted from each of the five test sets *P*1, *P*2, *P*3, *P*4 and *P*5.

Test Set	Train Set	Mean Sensitivity	STDEV
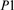	{ 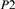 , 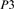 , 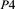 , 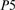 }	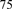	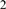
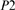	{ 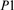 , 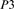 , 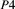 , 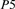 }	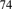	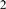
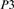	{ 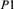 , 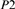 , 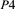 , 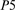 }	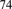	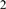
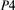	{ 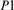 , 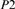 , 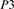 , 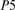 }	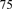	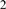
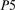	{ 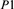 , 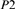 , 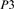 , 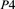 }	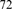	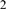
	Average	74	2

### Conclusion

We conclude that an *n*-gram Markov chain can be used to learn syntactic features of the Indus script that depend on the contiguity of signs. Our analysis shows that a quadrigram model appears to be sufficient for this purpose. We find a Zipf-Mandelbrot distribution for unigrams and unequal distributions in the frequencies of text beginners and text enders, which provides internal evidence for syntax in writing. Using a log-likelihood ratio test of association, we find significant sign pairs and triplets, which do not always correspond to high-frequency sign pairs and triplets. Using entropic measures we find that trigrams and quadrigrams make increasingly modest contributions to the overall correlations in the script. A bigram version of the model is used to suggest probable restorations of illegible signs from a corpus and a measure of model performance is provided using cross-validation. The combined results of our analysis, summarized in Table 6, along with our earlier work [12]–[15], indicate that the script has a rich syntax with an underlying logic in its structure which needs to be explored further. Our results provide evidence in favor of the linguistic hypothesis for the script but additional work is required to reach a conclusive verdict. To the best of our knowledge, our work represents the first use of the methods of *n*-gram analysis to an undeciphered script. We believe probabilistic methods hold considerable promise in elucidating syntax in undeciphered scripts. Inducing grammar and syntactic structures for the Indus script based on Markov chains is part of ongoing work.

**Table 6:** Major conclusions.

Sl. No	Test/Measure	Results	Fig./Table No.	Conclusions
1.	Zipf-Mandelbrot law	Best fit for a = 15.39, b = 2.59, c = 44.47 (95% Confidence interval)	Fig. 3	Small number of signs account for bulk of the data while a large number of signs contribute to a long tail.
2.	Cumulative frequency distribution	69 signs: 80% of EBUDS, 23 signs: 80% of Text Enders, 82 signs: 80% of Text Beginners	Fig. 4	Indicates asymmetry in usage of 417 distinct signs. Suggests logic and structure in writing.
3.	Bigram probability	Conditional probability matrix for EBUDS is strikingly different from the matrix assuming no correlations.	Fig. 5	Indicates presence of significant correlations between signs.
4.	Conditional probabilities of text beginners and text enders	Restricted number of signs follow frequent text beginners whereas large number of signs precede frequent text enders.	Fig. 7	Indicates presence of signs having similar syntactic functions.
5.	Log-likelihood significance test	Significant sign pairs and triplets extracted.	Tables 2, 3	The most significant sign pairs and triplets are not always the most frequent ones.
6.	Entropy	Random: 8.56; EBUDS: 6.68	Table 4	Indicates presence of correlations.
7.	Mutual information	Random: 0; EBUDS: 2.24	Table 4	Indicates flexibility in sign usage.
8.	Perplexity	Monotonic reduction as n increases from 1 to 5.	Table 1	Indicates presence of long range correlations, see also [12], [13].
9.	Sign restoration	Restoration of missing and illegible signs.	Figs. 11, 12	Model can restore illegible signs according to probability.
10.	Cross validation	Sensitivity of the bigram model = 74%	Table 5, Fig. 14	Model can predict signs with 74% accuracy.

## Materials and Methods

### Corpus

The corpus used for analysis is that of Mahadevan, compiled in 1977 [3], henceforth referred to as M77. It records 417 unique signs in 3573 lines of 2906 texts. The serial number of the signs used in this paper is as given in M77. As a convention followed in the present paper, the texts depicted by pictures are to be read from right to left, whereas the texts represented by just strings of sign numbers are to be read from left to right (see M77 for discussion on direction of texts). Moreover, throughout the paper, we have used *text beginners* to refer to the signs at the right extreme of texts depicted by pictures, and *text enders* to refer to the signs at the left extreme of texts depicted by pictures. In order to remove ambiguity, an Extended Basic Unique Data Set (EBUDS) is created by removing from the concordance all texts containing lost, damaged, illegible, or doubtfully read parts [12]. Texts spread across multiple lines are also removed. For texts that occur more than once, only a single copy is retained. Variations due to the archaeological context of the sites, stratigraphy, and type of object on which the texts are inscribed are, at present, not taken into account in the interests of retaining a reasonable sample size.

The reasons for discarding texts which are spread over multiple lines are twofold. First, it is not clear if each line of a multi-line text is to be treated as a single text having a continuity of sequence, or if it is to be regarded as separate text. Second, assuming a continuity of sequence, the order in which the texts are to be read across lines remains ambiguous [3].

**Figure 15:**
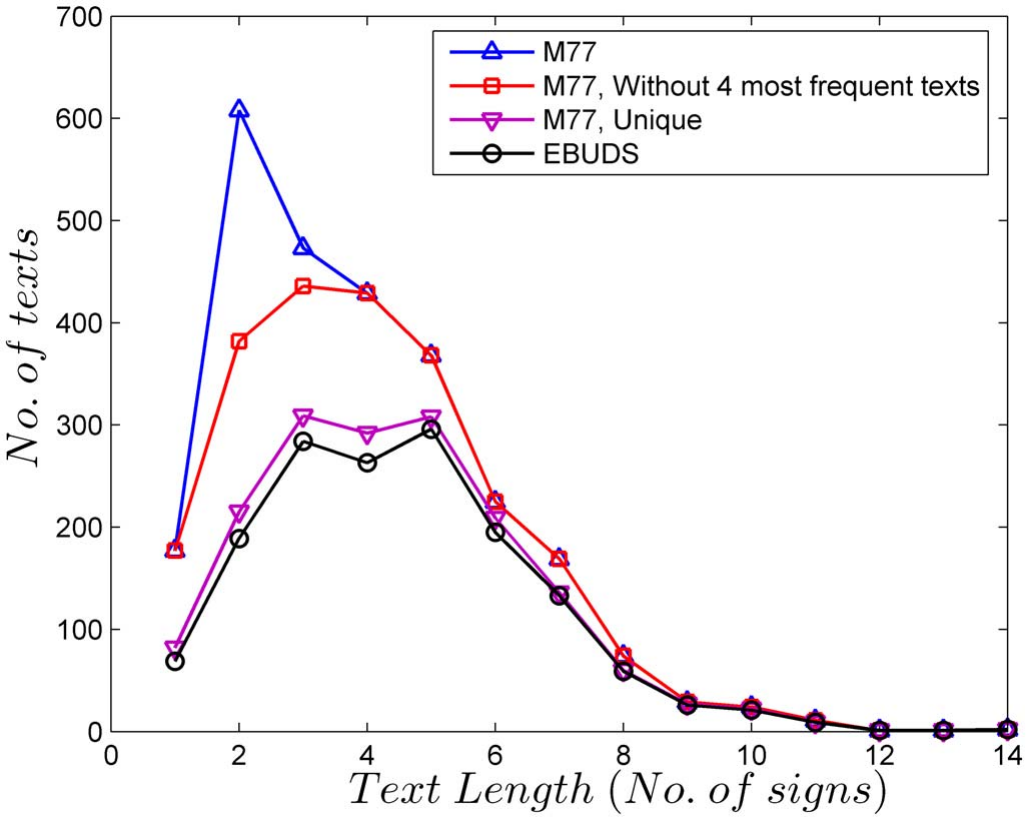
Text length distributions in the different corpora used in the analysis. The raw corpus (M77) contains four instances of outliers, texts of length *n* = 2 and *n* = 3 which occur in unusually large numbers. Keeping only single occurrences of these removes the sharp maximum around *n* = 2 in the raw corpus. The corpus free of the outliers is then reduced again to keep only unique occurrences of the texts. This gives the M77-unique corpus. Finally, damaged, illegible and multi-line texts are removed to give the EBUDS corpus. Texts of length *n* = 3 and *n* = 5 are most frequent in this corpus.

The EBUDS dataset contains 1548 texts. In EBUDS, 40 signs out of 417 present in the sign list of M77 do not make their appearance. Out of these removed 40 signs, one sign (374) appears 9 times, one sign (237) appears 8 times, two signs (282, 390) appear 3 times, three signs (324, 376, 378) appear twice and thirty-three signs appear only once in M77. Fig. 15 compares the distribution of texts lengths in various datasets and shows the effect of the removal of texts on the final dataset (EBUDS). We have already shown in our earlier work [12], that the relative frequency distribution of signs in EBUDS is comparable to M77 and hence EBUDS is a true representation of M77, with a reduction in total sign occurrences, but not in the percentage of total sign occurrences.

Other corpora include those of Parpola (see [4]) and Wells (see [5]). Preliminary results from a comparative statistical analysis of these corpora indicate that the major syntactic features are robust across these corpora.

### Probability of Sequences

We denote a sequence of *N* signs by 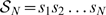, where each 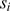 is one of the 417 possible Indus signs. Each of these *S_N_* is referred to as a text. The EBUDS corpus contains texts of maximum length *N* = 14. In the empirical analysis above, we have obtained frequency distributions for the signs *s_i_* by counting the number of times *c*(*s_i_*) that sign *s_i_* occurs in the corpus, and then dividing it by the total size of the corpus. This is identified with the *probability*, in the sense of maximum likelihood, of seeing the sign *s_i_* in a text,
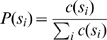
(11)In the absence of correlations, the joint probability that we see sign 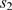 after sign 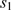 is independent of 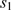, and is just the product of their individual probabilities
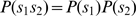
(12)Generalizing, the probability of the string 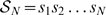 is simply a product of the individual probabilities
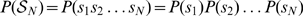
(13)In the absence of correlations, then, we have a scenario analogous to die throwing, where instead of 6 possible outcomes, we have 417 possible outcomes in each throw, and the 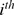 outcome has a probability P(*s_i_*). Each throw outputs a single sign, and the outcome of a throw is independent of all previous throws. In *N* throws, we generate a line of text *S_N_*. Our analysis shows that such an unigram model, where each outcome is independent of the previous outcome, is not adequate to model the EBUDS corpus. Thus it necessary to explicitly account for correlations, which can be done systematically using *n*-gram Markov chains.

### Information Theoretic Measures

The information theoretic measures, entropy *H* and the mutual information *I*, used to quantify the bigram correlations in the sequences are given below.
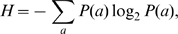
(14)

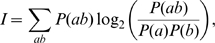
(15)


### Learning Markov Chains

**Figure 16:**
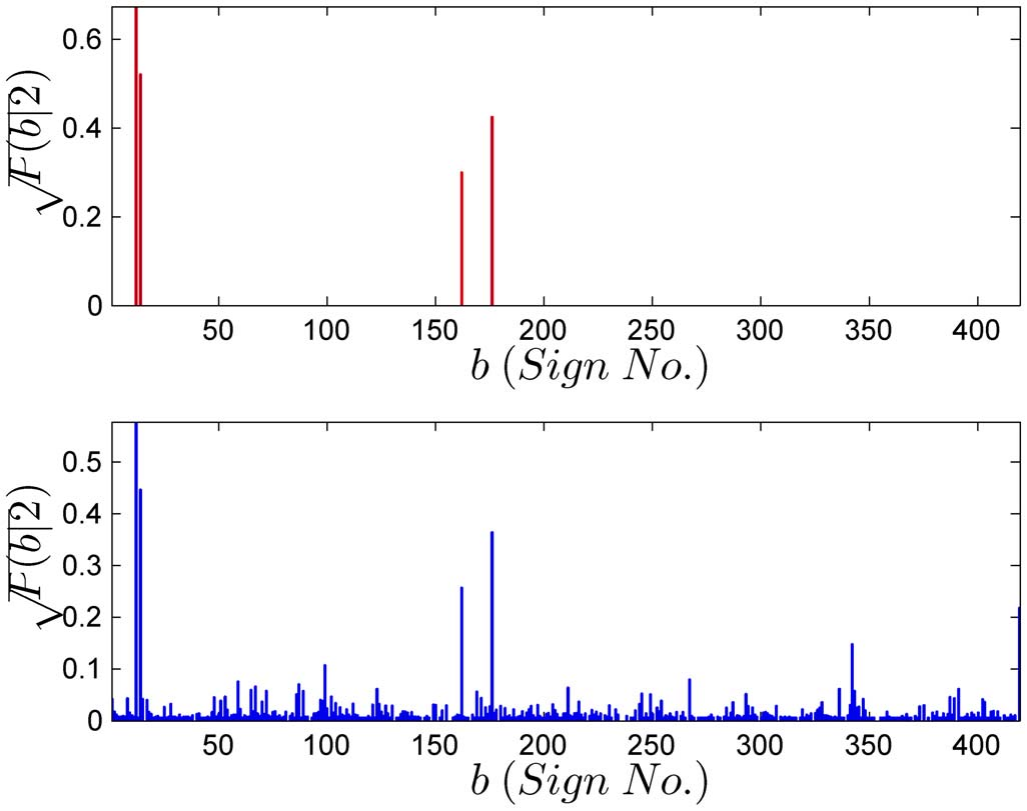
The conditional probability *P*(*b*|*a* = 2) from the maximum likelihood estimate (above) and from Witten-Bell smoothing (below). The maximum likelihood estimate assigns zero probabilities to unseen sign pairs and results in a non-ergodic Markov chain. The Witten-Bell smoothing algorithm reduces the probabilities of the seen sign pairs and distributes the reduction over unseen sign pairs. This gives an ergodic Markov chain. The square root of conditional probabilities are plotted in each case to highlight the probabilities of unseen sign pairs.

*n*-gram probabilities are obtained from counts. For single signs, the counts are unambiguous. However, for sign pairs, it is possible that a rare combination may not be present in a small sized corpus. The count, and the resulting probability estimate for that sign pair, then is zero. However, in the absence of reasons to the contrary, common sense dictates that no sign pair should have a strictly zero probability. This intuition is quantified by various rules which remove probability weight from seen sign pairs and distribute it to sign pairs which have never been seen, but are nonetheless not impossible. Common amongst such “smoothing” rules are Laplace's add-one rule, a method developed by Turing and Good in breaking the Enigma code, and a more recent algorithm due to Witten and Bell [26]. Here, we use the Witten-Bell algorithm to smooth our *n*-gram models. In Fig. 16 we show the estimate of the probability of a sign being followed by sign 2, 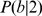 before smoothing and after Witten-Bell smoothing. In above panel, the only non-zero probabilities are those corresponding to signs 12,14,162 and 176. These probabilities sum to one, indicating that other sign pairs are impossible. The Witten-Bell smoothing algorithm restores a finite, but small probability to the unseen sign pairs, ensuring again that all probabilities sum to one. Apart from being a more reasonable way of estimating probabilities from counts, it also ensures that the resulting Markov chain is ergodic. A Markov chain is ergodic if it is possible to reach every state from every state (not necessarily in one move) and is aperiodic. An ergodic Markov chain is essential in such applications, since otherwise, probabilities of all strings containing unseen *n*-grams vanish.

Smoothing is not the only way of correcting for unseen *n*-grams. Another method, called backoff uses probabilities of (*n*−1) -grams to estimate *n*-gram probabilities. Thus, the probability of unseen trigrams can be estimated from that of seen bigrams and unigrams. Here, we used the Katz backoff algorithm to estimate bigram, trigram and quadrigram probabilities when appropriate.

The estimation of *n*-gram probabilities from *n*-gram counts is called learning. A learned *n*-gram model can then be tested to see if it produces *n*-grams in the corpus. To avoid circularity, the corpus is usually divided into a training set, from which the probabilities are estimated, and a test set, on which the model is evaluated. There are several standard measures for evaluating the goodness of an *n*-gram model. Here, we use a standard measure, the perplexity, which is related to the information theoretic measure, the cross-entropy. We also do a cross-validation test using standard procedures.

Finally, we need tests of association to ascertain the significance of sign pairs which appear more or less frequently than what would be predicted by the Bernoulli scheme model. For this, we use a log-likelihood ratio test, testing the null hypothesis that there is no association between sign pairs [24]. We leave more sophisticated statistical analyses [27] of significance of association to future work.

Markov chain learning was implemented using the SRILM toolkit [28] while the NSP [29] toolkit was used for the log-likelihood ratio test of association.
